# Modeling renal autoregulation in a hemodynamic, first‐trimester gestational model

**DOI:** 10.14814/phy2.15484

**Published:** 2022-10-06

**Authors:** Maaike van Ochten, Berend E. Westerhof, Marc E. A. Spaanderman, Tim A. J. Antonius, Joris van Drongelen

**Affiliations:** ^1^ Department of Gynecology and Obstetrics Radboud University Medical Center Nijmegen The Netherlands; ^2^ Department of Gynecology and Obstetrics Maastricht University Medical Center Maastricht The Netherlands; ^3^ Division of Neonatology, Department of Perinatology Radboud University Medical Center, Radboud Institute for Health Sciences, Amalia Children's Hospital Nijmegen The Netherlands; ^4^ Department of Pulmonary Medicine Amsterdam University Medical Centers, Vrije Universiteit Amsterdam, Amsterdam Cardiovascular Sciences Amsterdam The Netherlands

**Keywords:** hemodynamic model, myogenic response, pregnancy, renal autoregulation, tubuloglomerular feedback

## Abstract

The maternal cardiovascular system, led by renal volume regulatory responses, changes during pregnancy to ensure an adequate circulation for fetal development and growth. Circulatory maladjustment predisposes to hypertensive complications during pregnancy. Mathematical models can be used to gain insight in the gestational cardiovascular physiology. In this study, we developed an accurate, robust, and transparent model for renal autoregulation implemented in an existing circulatory gestational model. This renal autoregulation model aims to maintain steady glomerular pressure by the myogenic response, and glomerular filtration rate by tubuloglomerular feedback, both by inducing a change in the radius, and thus resistance, of the afferent arteriole. The modeled response of renal blood flow and the afferent arteriole following blood pressure increase were compared to published observations in rats. With solely the myogenic response, our model had a maximum deviation of 7% in change in renal blood flow and 7% in renal vascular resistance. When both the myogenic response and tubuloglomerular feedback were concurrently activated, the maximum deviation was 7% in change in renal blood flow and 5% in renal vascular resistance. These results show that our model is able to represent renal autoregulatory behavior comparable to empirical data. Further studies should focus on extending the model with other regulatory mechanisms to understand the hemodynamic changes in healthy and complicated pregnancy.

## Graphical abstract

We developed a renal autoregulation model implemented in a first trimester hemodynamic model. Simulation results of renal blood flow, afferent resistance and afferent radius as a response to an increase in blood pressure are in line with observations in empirical data.

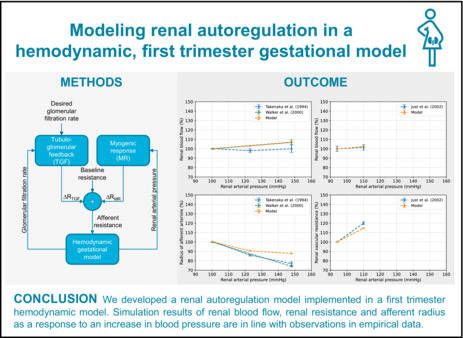

## 1 INTRODUCTION

During pregnancy, the maternal cardiovascular system changes to ensure adequate circulation for fetal development and growth. These alterations are most profound in the first trimester and include lowering of the arterial blood pressure, increase in plasma volume, cardiac output, and heart rate in response to a decrease in total peripheral vascular resistance (de Haas et al., 2022; Lopes van Balen et al., 2019). In these changes, renal physiology is involved as it plays a pivotal role in the volume regulatory adjustments, most likely triggered by a decreased renal vascular resistance. Glomerular filtration rate (GFR) increases by 50% and renal plasma flow increases up to 80% (Cheung & Lafayette, 2013; Dunlop, 1981; Lopes van Balen et al., 2019; Sanghavi & Rutherford, 2014). Glomerular pressure does not increase despite the rise in renal flow, which is the result of proportional reduction in both the afferent and efferent arteriolar resistances (Baylis, 1994). Total blood plasma volume increases by almost 1.5 L (de Haas et al., 2017; Sanghavi & Rutherford, 2014), the kidney itself increases up to 30% in volume (Cheung & Lafayette, 2013). Even the renal autoregulation is adjusted as the increase in GFR and renal flow is paralleled by a reset in tubuloglomerular feedback (TGF) that allows these elevated flows to be recognized as normal throughout gestation (Baylis, 1994; Ogueh et al., 2011; Woods et al., 1987). Inability to adapt to these physiological gestational changes during the first trimester is associated with subsequent cardiovascular complications in the second and third trimesters (Lopes van Balen et al., 2013).

Hypertensive disorders affect up to 10% of the pregnancies worldwide and are preceded by hemodynamic maladjustments resulting in fetal, neonatal, and maternal morbidity and mortality (Arnott et al., 2020; Khan et al., 2006; Sanghavi & Rutherford, 2014; Say et al., 2014). In hypertensive complicated pregnancies, kidney function is impaired, which implies a malfunctional and insufficient tubuloglomerular feedback mechanism (Lopes van Balen et al., 2019). In addition, a disturbed myogenic response (MR) may fail to protect glomeruli from elevated blood pressure and renal vascular damage may ensue (Baylis, 1994; Kublickas et al., 1996). However, the interaction between the systemic and renal autoregulatory adjustments in pregnancy are still poorly understood. To counsel women on the risks of hypertensive disease in a future pregnancy, it is of importance to develop an accurate simulation model to predict associated maternal and offspring risk in the individual.

Integrated mathematical models accounting for the whole‐body hemodynamic changes during pregnancy can help to simulate and better understand the interplay of underlying mechanisms of healthy and unhealthy cardiovascular adjustments and the subsequent effects on pregnancy outcomes (Euliano et al., 1997; Goodwin et al., 2004; van Meurs & Antonius, 2018). Our group works on a renal simulator implemented in a mathematical whole‐body circulatory environment. The first step in this research will focus on modeling the local, short‐term renal autoregulatory mechanism. Therefore, the aim of this study was to stepwise develop and validate an accurate, robust, and transparent model for renal autoregulation during pregnancy.

## 2 METHODS

The MR and TGF are modeled separately and then implemented in the complete hemodynamic model of first‐trimester pregnancy. The modeling approach in this study consists of the following steps: designing a conceptual model, converting this to a mathematical model, for which parameters are estimated, and finally, validating the modeled system.

### 2.1 Model description

The whole‐body hemodynamic model used in this study is adapted from the lumped compartment model as proposed by van Meurs and Antonius in combination with the model proposed by Goodwin et al. (Goodwin et al., 2004; van Meurs & Antonius, 2018). The model consists of “compartments” and “connectors”: compartments are defined as compliances containing volume, which, in turn, are combined by connectors that do not contain volume but can be described as resistances. The hemodynamic model can be described by the following basic equations:

(1) $$P\left(t\right)=E*\left(V\left(t\right)-V_{0}\right)$$

(2) $$q_{\text{in}}\left(t\right)=\frac{1}{R}*\left(P_{1}\left(t\right)-P_{2}\left(t\right)\right)$$

(3) $$\frac{\mathrm{dV}\left(t\right)}{\mathrm{dt}}=q_{\text{in}}\left(t\right)-q_{\text{out}}\left(t\right)$$

Equation 1 describes how pressure (*P(t)*) depends on the elastance (*E*) and volume of the compartment, where *V*_*0*_ is the unstressed volume and *V(t)* is the total volume. The unstressed volume is the volume that can reside in a compartment without stretching the walls. The flow (*q*_*in*_ *(t)*) from one compartment to another, resulting from a pressure gradient is described in Equation 2, where *R* is the resistance of the connector between the two compartments. Equation 3 describes the volume change in a compartment as a result of incoming and outgoing flow. This equation also closes the hemodynamic loop, as the input volume for Equation 1 is generated.

**FIGURE 1:**
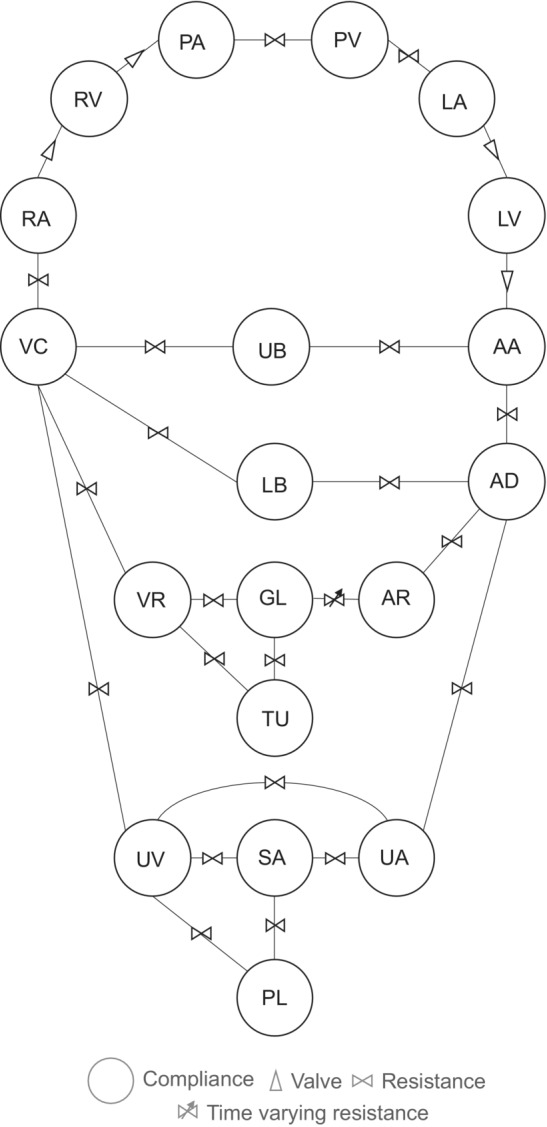
The hemodynamic lumped compartment model of a pregnant woman. The model consists of the pulmonary veins (PV), left atrium (LA), left ventricle (LV), ascending aorta (AA), descending aorta (AD), upper body (UB), lower body (LB), renal arteries (AR), glomerulus (GL), renal tubule (TU), renal veins (VR), uterine arteries (UA), spiral arteries (SA), placenta (PL), uterine veins (UV), vena cava (VC), right atrium (RA), right ventricle (RV), and pulmonary arteries (PA).

The current gestational model consists of a pulmonary and systemic circulation; the conceptual model can be seen in Figure 1. The heart is modeled as four separate compartments with time‐varying elastances: the right atrium (RA), right ventricle (RV), left atrium (LA), and left ventricle (LV). The elastances of these compartments vary over the cardiac cycle and depend on the end‐systolic pressure‐volume relations, representing myocardial contractility and the end‐diastolic pressure‐volume relationship, representing diastolic myocardial stiffness (Maksuti et al., 2016; Senzaki et al., 1996; Stergiopulos et al., 1996). The pulmonary arteries (PA) and pulmonary veins (PV) are modeled separately and implemented between the RV and LA. Valves are modeled as connectors with an infinite backward resistance, to prevent backward flow.

The systemic circulation was originally modeled by five compliances: the ascending aorta (AA), descending aorta (AD), upper body (UB), lower body (LB), and caval vein (VC). The model has been extended by splitting the LB in three different parts: the kidneys, uterus, and remaining LB. The uterus is defined by four compliances: the uterine arteries (UA), spiral arteries (SA), maternal placenta (PL), and the uterine veins (UV). The kidneys are modeled as a single, lumped nephron which consists of two compartments: the glomerulus (GL) and tubule (TU). In addition, the renal arteries (AR) and veins (VR) are added to the model. For the current study, the renal autoregulation, consisting of the MR and TGF, ais implemented, which will be detailed later.

#### 2.1.1 Model parameters

The model parameters for the hemodynamic model are the elastance (mmHg/L), starting volume (L), and unstressed volume (L) for each compartment, the resistance (mmHg*s/L) for each connector and the heart rate (bpm). The hemodynamic changes that occur during the first trimester of pregnancy are taken into account in this model. These include increased starting volume and increased volumes of RA, RV, LA, LV, and the compartments belonging to the kidneys and uterus as compared to the non‐pregnant condition (Cheung & Lafayette, 2013; Del Prado et al., 2020; Sanghavi & Rutherford, 2014; Song et al., 2015). Model parameters are based on prior studies (BioGears, 2018; Goodwin et al., 2004; van Meurs & Antonius, 2018) and were adjusted to reach gestational values. As from 4 to 8 weeks gestation, tremendous gestational changes and resetting of most systems already take place, the goal is to reach physiological hemodynamic values of an 8‐week pregnant condition, which are retrieved from original empirical data (Spaanderman et al., 2000). Especially the input parameters for the renal connectors and compartments are evaluated, as these are most important for the renal autoregulation model. Total renal resistance, consisting of the resistances AD_AR, AR_GL, GL_VR, GL_TU, TU_VR, and VR_VC (Figure 1), is estimated with the goal to obtain a renal blood flow (RBF) of about 1 L/min (Spaanderman et al., 2000). The value of each individual resistance is estimated based on ratios as proposed by the BioGears open‐source engine and Guyton (BioGears, 2018; Guyton & Hall, 2006). The goal is to reach a GFR of 0.149 L/min and pressures in the renal compartments (AR, GL, TU, and VR) in agreement with the physiological conditions at 8‐week gestational age (Table A3). The resistances AA_UB, AD_LB, UB_VC, and LB_VC were adjusted to obtain a cardiac output between 5.7 and 6 L/min (Spaanderman et al., 2000).

#### 2.1.2 Renal autoregulation model

**FIGURE 2:**
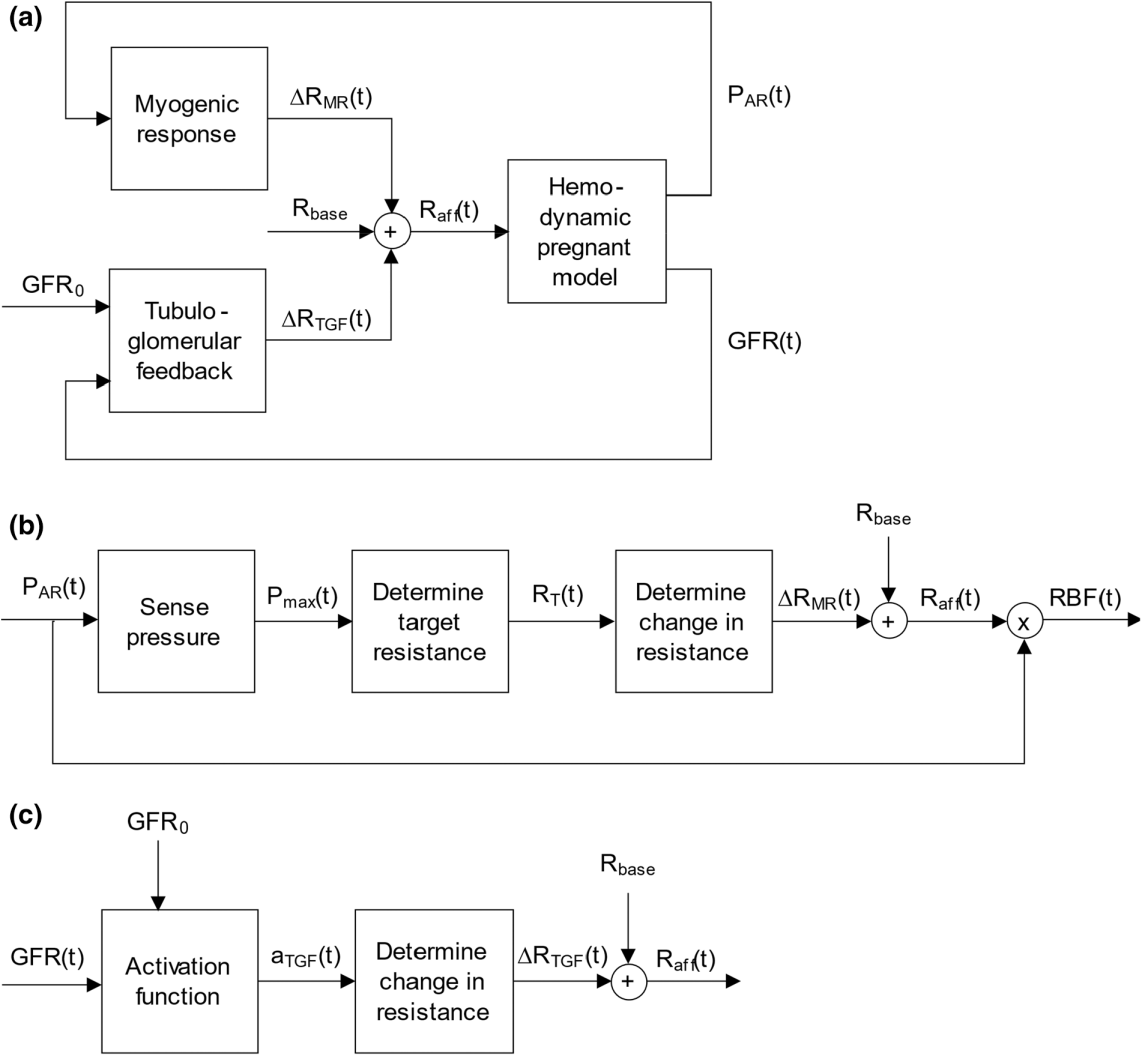
Block diagrams of the complete renal autoregulation model, MR model, and TGF model. (a) Renal autoregulation model. Pressure in the renal artery (P_AR_) induces a change in afferent resistance through the myogenic response. Glomerular filtration rate (GFR) induces a change in afferent resistance through the tubuloglomerular feedback. (b) Myogenic response model. (c) TGF model. (P_AR_, pressure in renal artery compartment; P_max_, maximal pressure; R_T_, target resistance; ∆R_MR_, change in resistance by myogenic response; R_base_, baseline resistance; R_aff_, afferent arteriolar resistance; RBF, renal blood flow.

The renal autoregulation model consists of the MR and TGF. A block diagram with the input and output variables for each mechanism and their place in the gestational hemodynamic model is shown in Figure 2a. The MR and TGF are modeled separately and will be combined to alter the resistance of connector AR_GL, which represents the afferent arteriole. The changes in resistance determined by the MR (*dR*_*MR*_ *(t)*) and TGF (*dR*_*TGF*_ *(t)*) are added to the baseline resistance (*R*_*base*_) to set the new resistance:

(4) $$R_{\mathrm{AR}\_\mathrm{GL}}\left(t\right)=R_{\text{base}}+{\mathrm{dR}}_{\mathrm{MR}}\left(t\right)*g_{\mathrm{MR}}+{\mathrm{dR}}_{\mathrm{TGF}}\left(t\right)*g_{\mathrm{TGF}}\;$$

The control gains *g*_*MR*_ and *g*_*TGF*_ determine the amplitude of the effect of the autoregulatory components. These gains are determined so that blood pressures and blood flows remain within physiological ranges when renal autoregulation is active. The new resistance will then be used in the gestational hemodynamic model to determine pressure and flows according to the model equations as defined before.

#### 2.1.3 Myogenic response

The renal MR describes the effect on the diameter and thus resistance of the afferent arteriole induced by a change in blood pressure. The implemented renal myogenic response is based on the model as described by Williamson et al. (Williamson et al., 2008). A schematic overview of our proposed MR model is shown in Figure 2b. The sensed vascular blood pressure in the AR (*P*_*AR*_ *(t)*) is determined as the maximum of *P(t)* over an interval of time between *t – δ*_*2*_ and *t – δ*_*1*_, where *δ*_*1*_ corresponds to the delay between an abrupt increase in blood pressure and the onset of vasoconstriction and *δ*_*2*_ to the delay between an abrupt decrease in blood pressure and the onset of vasodilation. Maximal pressure is used because it has been shown that the MR is most sensitive to changes in systolic blood pressure (Equation 5) (Williamson et al., 2008).

(5) $$\begin{array}{c} P_{\mathrm{AR}}\left(t\right)=\max\;P\left(\tau \right)\;\text{for}\;\tau \in \left[t-\delta_{2},,t-\delta_{1}\right] \end{array}$$

Changes in *P*_*AR*_ *(t)* result in a change in conductance of the afferent arteriole. First, *P*_*AR*_ *(t)* is translated to a target conductance *c*_*T*_ *(t)*. This target conductance is determined so that it agrees with the autoregulation curves describing the relation between conductance and systolic blood pressure and RBF and systolic blood pressure. *c*_*T*_ *(t)* is determined by the constants *p*_*0*_, *p*_*1*_, *q*_*0*_, and *k* and the variables *P*_*AR*_ *(t)* and *P*_*VC*_ *(t)* as described in Equation 6,^1^ where *P*_*VC*_ *(t)* is the pressure in the VC. *p*_*0*_ and *p*_*1*_ are the renal blood pressures between which the myogenic response affects the conductance, *q*_*0*_ is the desired renal blood flow. *k* reflects the relative fractional change in flow resulting from a change in pressure. We included *P*_*VC*_ *(t)* in our equation so that the pressure gradient over the renal compartments is used to determine the desired conductance.

(6) $$\begin{array}{c} c_{T}\left(t\right)\\ =\left\{\begin{array}{c} \left(\frac{q_{0}}{p_{1}-P_{\mathrm{VC}}\left(t\right)}\right)\left(1+\frac{k\left(p_{1}-p_{0}\right)}{p_{0}}\right)\;\;\;\;\;\;\;\;\;\;\text{if}\;P_{\mathrm{AR}}\left(t\right)>p_{1}\;\;\;\\ \left(\frac{q_{0}}{P_{\mathrm{AR}}\left(t\right)-P_{\mathrm{VC}}\left(t\right)}\right)\left(1+\frac{k\left(P_{\mathrm{AR}}\left(t\right)-p_{0}\right)}{p_{0}}\right)\;\;\;\;\;\text{if}\;p_{0}\leq P_{\mathrm{AR}}\left(t\right)\leq p_{1}\;\;\;\;\;\;\;\;\;\\ \left(\frac{q_{0}}{p_{0}-P_{\mathrm{VC}}\left(t\right)}\right)\;\;\;\;\;\;\;\;\;\;\;\;\;\;\;\;\;\;\;\;\;\;\;\;\;\;\;\;\;\;\;\;\;\;\;\;\;\;\;\;\;\;\;\;\;\;\text{if}\;P_{\mathrm{AR}}\left(t\right)<p_{0}\;\;\;\;\;\;\;\;\;\;\;\;\;\;\;\;\;\; \end{array}\right. \end{array}$$

Since our model equations use resistance instead of conductance as an input parameter, conductance is converted to resistance by Equation 7.

(7) $$R=c^{-1}$$

The determined target resistance is the total renal resistance from compartment AR to VC, however, the intention is to only affect the resistance of the connector representing the afferent arteriole (AR_GL) by the MR model. The other resistances of the renal connectors (GL_VR, GL_TU, TU_VR, and VR_VC) remain constant. Therefore, the target resistance of solely the afferent arteriole is determined according to Equation 8.

(8) $$R_{T}\left(t\right)=c_{T}{\left(t\right)}^{-1}-{\left(\frac{1}{R_{\mathrm{GL}\_\mathrm{VR}}}+\frac{1}{R_{\mathrm{GL}\_\mathrm{TU}}+R_{\mathrm{TU}\_\mathrm{VR}}}\right)}^{-1}-R_{\mathrm{VR}\_\mathrm{VC}}$$

The calculated target resistance is compared to the baseline resistance to determine the desired total change in resistance, *ΔR* (Equation 9).

(9) $$∆R\left(t\right)=R_{T}\left(t\right)-R_{\text{base}}$$

The change in resistance per time step as a result of the myogenic response (*dR*_*MR*_ *(t)/dt*) is described in Equation 10. Here, *R(t)* is the vascular resistance at time *t* and $\tau_{1}$ and $\tau_{2}$ are the time constants for vasoconstriction and vasodilation, respectively.

(10) $$\begin{array}{c} \frac{{\mathrm{dR}}_{\mathrm{MR}}\left(t\right)}{\mathrm{dt}}\\ =\left\{\begin{array}{c} \frac{1}{\tau_{1}}*\left(-{\mathrm{dR}}_{\mathrm{MR}}\left(t-\mathrm{dt}\right)+∆R\left(t\right)\right)+\frac{dR_{\mathrm{MR}}\left(t-\mathrm{dt}\right)}{\mathrm{dt}}\;\text{if}\;R\left(t\right)>R_{T}\left(t\right)\\ \frac{1}{\tau_{2}}*\left(-{\mathrm{dR}}_{\mathrm{MR}}\left(t-\mathrm{dt}\right)+∆R\left(t\right)\right)+\frac{dR_{\mathrm{MR}}\left(t-\mathrm{dt}\right)}{\mathrm{dt}}\;\text{if}\;R\left(t\right)\leq R_{T}\left(t\right) \end{array}\right. \end{array}$$

Most model parameters for the MR are taken from published data, only parameter *k* had to be estimated. This parameter affects the slope of the autoregulatory curve defined by Equation 6. To determine the value of *k*, we compared our autoregulatory curve to measurements performed in the literature. In their study, Hayashi et al. measured the effect of increased renal blood pressure on the radius of the afferent arteriole in hydronephrotic Wistar–Kyoto (WKY) rats (*n* = 7) (Hayashi et al., 1989). In order to compare our results to theirs, the calculated resistance was converted to radius (*r)*, using that resistance is inversely proportional to radius to the fourth power ($R\sim \frac{1}{r^{4}}$). The change in radius at a systolic renal pressure of 80 mmHg is set as 0%, as the afferent arteriole is then maximally dilated (Boron & Boulpaep, 2012a; Rennke & Denker, 2006; Silverthorn, 2013). The value of *k* was chosen so that a change in radius of minus 20% was obtained at a renal systolic pressure of 180 mmHg (Hayashi et al., 1989).

#### 2.1.4 Tubuloglomerular feedback

TGF affects the resistance of the afferent arteriole if the macula densa senses a change in GFR. In our model, the flow from GL to TU reflects the GFR. A schematic overview of our TGF model is shown in Figure 2c. A linear activation function (Equation 11) is used to determine the change in resistance. The activation factor (*a*_*TGF*_) is defined as the difference between the operating point (*op*_*GFR*_) and the sensed GFR. The minimal and maximal activation factor are determined by the threshold (*th*_*GFR*_) and saturation (*sa*_*GFR*_). *δ*_*3*_ reflects the delay between a change in GFR at the glomerular side and the moment this change is measured by the juxtaglomerular apparatus. The activation factor *a*_*TGF*_ is used to determine the change in resistance per time step caused by tubuloglomerular feedback (*dR*_*TGF*_ *(t)/dt*), as described in Equation 12. Here, $\tau_{3}$ and $\tau_{4}$ are the time constants for vasoconstriction and vasodilation, respectively.

(11) $$\begin{array}{c} a_{\mathrm{TGF}}\left(t\right)=\\ \left\{\begin{array}{c} {\mathrm{sa}}_{\mathrm{GFR}}-{\mathrm{op}}_{\mathrm{GFR}}\;\;\;\text{if}\;\mathrm{GFR}\left(t-\delta_{3}\right)\geq {\mathrm{sa}}_{\mathrm{GFR}}\;\;\\ \mathrm{GFR}\left(t-\delta_{3}\right)-{\mathrm{op}}_{\mathrm{GFR}}\;\;\;\;\;\text{if}\;\;{\mathrm{th}}_{\mathrm{GFR}}<\mathrm{GFR}\left(t-\delta_{3}\right)<{\mathrm{sa}}_{\mathrm{GFR}}\;\;\;\;\;\\ {\mathrm{th}}_{\mathrm{GFR}}-{\mathrm{op}}_{\mathrm{GFR}}\;\;\text{if}\;\mathrm{GFR}\left(t-\delta_{3}\right)\leq {\mathrm{th}}_{\mathrm{GFR}}\;\;\; \end{array}\right. \end{array}$$

(12) $$\begin{array}{c} \frac{dR_{\mathrm{TGF}}\left(t\right)}{\mathrm{dt}}\;\\ =\left\{\begin{array}{c} \frac{1}{\tau_{3}}*\left(-dR_{\mathrm{TGF}}\left(t-\mathrm{dt}\right)+a_{\mathrm{TGF}}\left(t\right)\right)+\frac{dR_{\mathrm{TGF}}\left(t-\mathrm{dt}\right)}{\mathrm{dt}}\;\text{if}\;a_{\mathrm{TGF}}\left(t\right)>0\\ \frac{1}{\tau_{4}}*\left(-dR_{\mathrm{TGF}}\left(t-\mathrm{dt}\right)+a_{\mathrm{TGF}}\left(t\right)\right)+\frac{dR_{\mathrm{TGF}}\left(t-\mathrm{dt}\right)}{\mathrm{dt}}\;\text{if}\;\;a_{\mathrm{TGF}}\left(t\right)\leq 0 \end{array}\right. \end{array}$$

The model parameters for the activation function were determined where *op*_*GFR*_ was based on measurements performed in earlier research (Spaanderman et al., 2000) and *th*_*GFR*_ and *sa*_*GFR*_ were estimated based on the functional purpose of the TGF system. We determined what the GFR of our model would be without renal autoregulation at a mean arterial pressure (MAP) of 80 mmHg and 180 mmHg and set these values as the *th*_*GFR*_ and *sa*_*GFR*_, since the goal of TGF is to maintain GFR in the blood pressure range from 80 to 180 mmHg (Boron & Boulpaep, 2012a; Rennke & Denker, 2006; Silverthorn, 2013).

The gain (*g*_*TGF*_) was determined by comparing our model results to measurements performed by Walker et al. (2000). They observed the change in RBF and radius of an afferent arteriole induced by an increase in blood pressure from 100 to 148 mmHg in an isolated nephron, where they looked into the effect of solely the MR, and the MR and TGF together. The same blood pressure step was imposed on the gestational hemodynamic model and the gain was determined so that the model output was in agreement with the observations from Walker et al. (2000).

### 2.2 Validation

The behavior of the renal autoregulation model was compared to previous findings in animal experiments. First, the model with only the myogenic response was compared to observations in rat studies by evaluating the change in radius of the afferent arteriole over a range of blood pressures (Just & Arendshorst, 2003; Loutzenhiser et al., 2002; Walker et al., 2000; Ren et al., 2010; Takenaka et al., 1994). Furthermore, the change in RBF and afferent arteriolar radius and renal vascular resistance generated by the complete autoregulation model after an increase in blood pressure was compared to earlier observations in rats (Just & Arendshorst, 2003; Takenaka et al., 1994). As we did not find any published data on the TGF solely, we validated the results of the TGF model in combination with the MR model. Values from previous studies are presented as mean ± SE. The performance of our model was classified based on the deviation of our model compared to published data. A deviation of less than 10% is considered very good, 10–20% good, 20–30% fair, and more than 30% poor (Antwi et al., 2020; Hanley & McNeil, 1982).

#### 2.2.1 Sensitivity analysis

To investigate to what extent the parameters *k* and *g*_*TGF*_ influence the results, a one‐at‐the‐time sensitivity analysis was performed. Parameters *k* and *g*_*TGF*_ were separately decreased and increased by 10% and 20%. We evaluated the influence of these parameters on the results of the autoregulation model.

## 3 RESULTS

### 3.1 Model parameters

**FIGURE B1:**
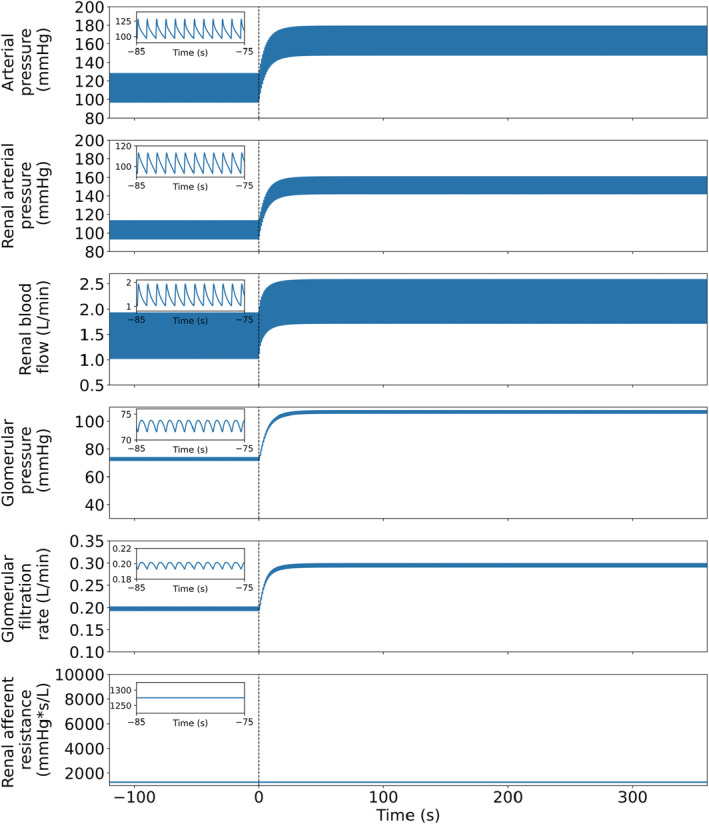
The dynamic behavior of renal arterial pressure, glomerular pressure, renal blood flow, glomerular filtration, and renal afferent resistance without renal autoregulation. At time *t* = 0 s (dashed line), mean renal arterial pressure is elevated from 100 to 148 mmHg. The subplots for the flows and pressures show small oscillations in these variables which are caused by heart rate.

Values for volumes, elastances, and resistances are presented in Appendix A (Table A1 and A2). The resulting blood flows, blood pressures, and their target values can be found in Appendix A in Table A3 and A4. Based on human data, heart rate was set to 69 beats per minute and the resulting cardiac output was equal to 5.8 L/min, corresponding to a stroke volume of 84 ml (Spaanderman et al., 2000). With these central hemodynamic characteristics, the goal to obtain an RBF of 1 L/min and a GFR of 0.149 L/min was reached. Also, the other modeled pressures and flows were within acceptable ranges of the target values. Response of renal pressures and flows to an increase in blood pressure without any interaction of renal autoregulation can be found in Appendix B (Figure B1).

### 3.2 Renal autoregulation model

#### 3.2.1 Myogenic response

**FIGURE 3:**
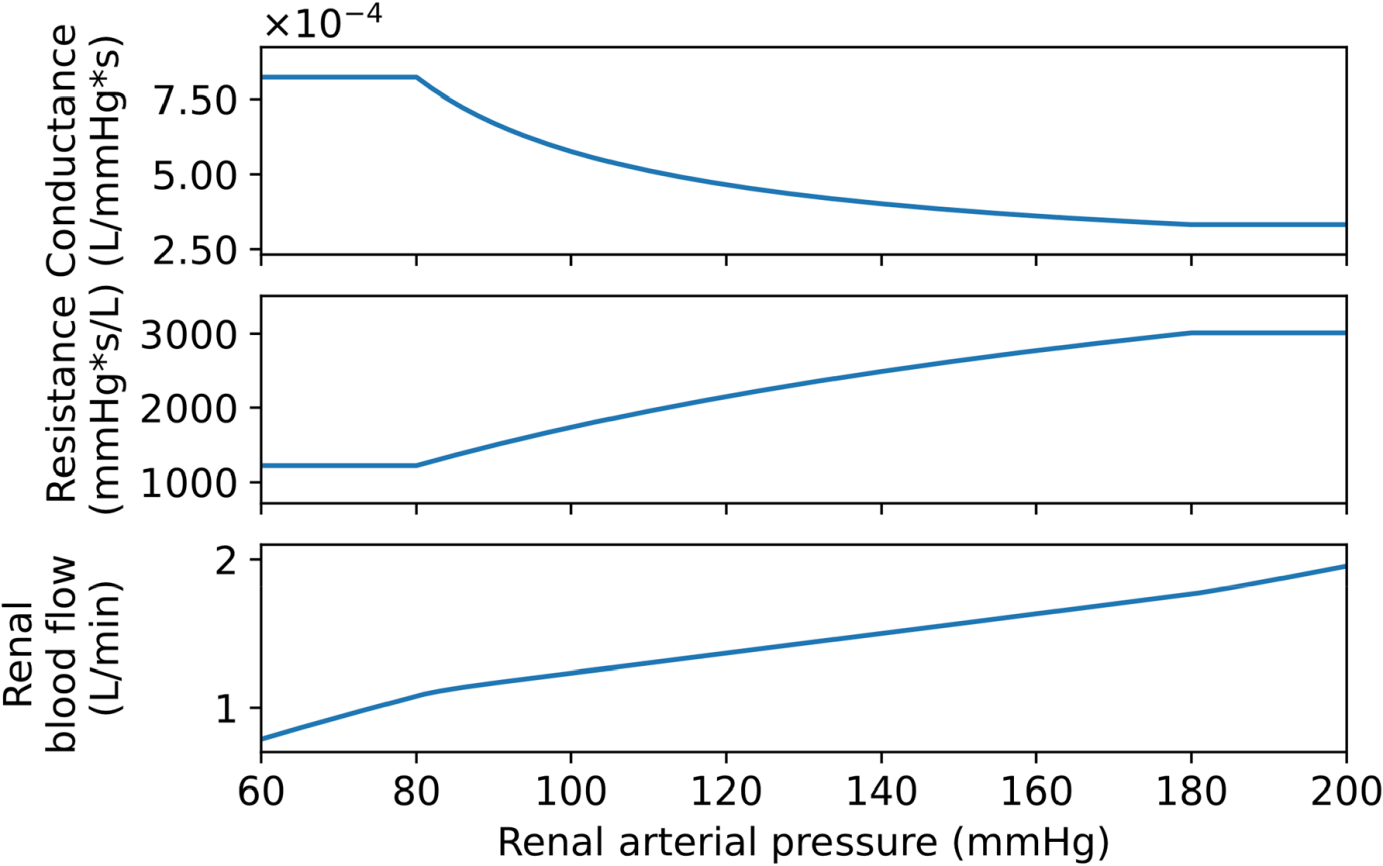
The autoregulatory curve for the myogenic response model.

**FIGURE B2:**
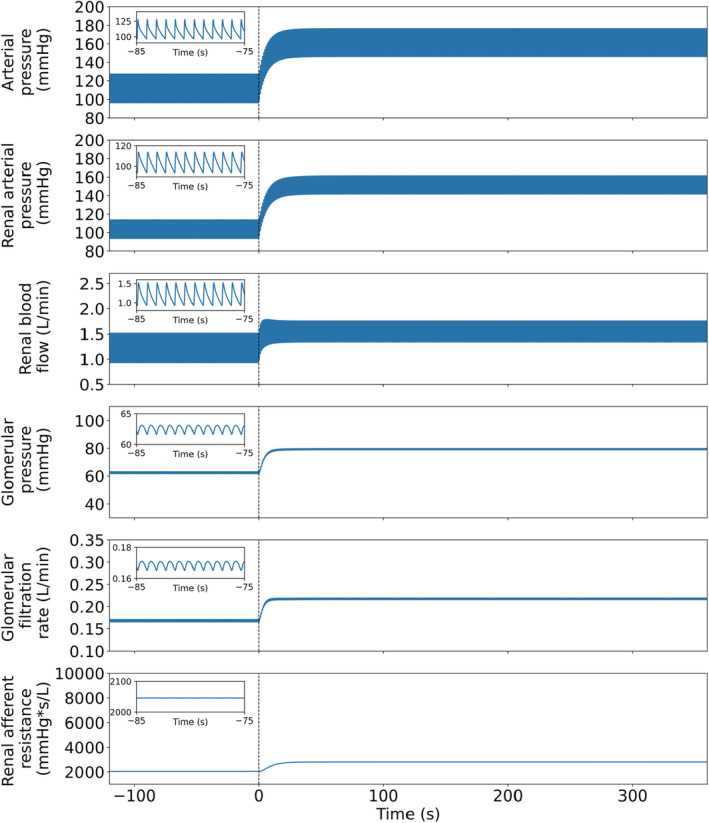
The dynamic behavior of renal arterial pressure, glomerular pressure, renal blood flow, glomerular filtration, and renal afferent resistance with renal autoregulation by solely the MR. at time *t* = 0 s (dashed line), mean renal arterial pressure is elevated from 100 to 148 mmHg. The subplots for the flows and pressures show small oscillations in these variables which are caused by heart rate.

The myogenic model parameters were set to *p*_*0*_ = 80 mmHg, *p*_*1*_ = 180 mmHg and *q*_*0*_ = 0.018 L/s (Boron & Boulpaep, 2012a; Rennke & Denker, 2006; Silverthorn, 2013; Spaanderman et al., 2000). The time constants and delays were set to $\tau_{1}$ = 4 s, $\tau_{2}$ = 5.3 s, *δ*_1_ = 0.3 s, and *δ*_2_ = 1.2 s (Williamson et al., 2008). Parameter *k* was set to 0.5, as this value best approximated our goal of a decrease in afferent radius by 20% at a renal pressure of 180 mmHg. As the goal of the change in radius was already reached by only changing *k*, the gain was set to *g*_*MR*_ = 1. The autoregulatory curve (Equation 6) with the set values is displayed in Figure 3. Response of renal pressures and flows to an increase in blood pressure and regulated by the myogenic response can be found in Appendix B (Figure B2).

**FIGURE 4:**
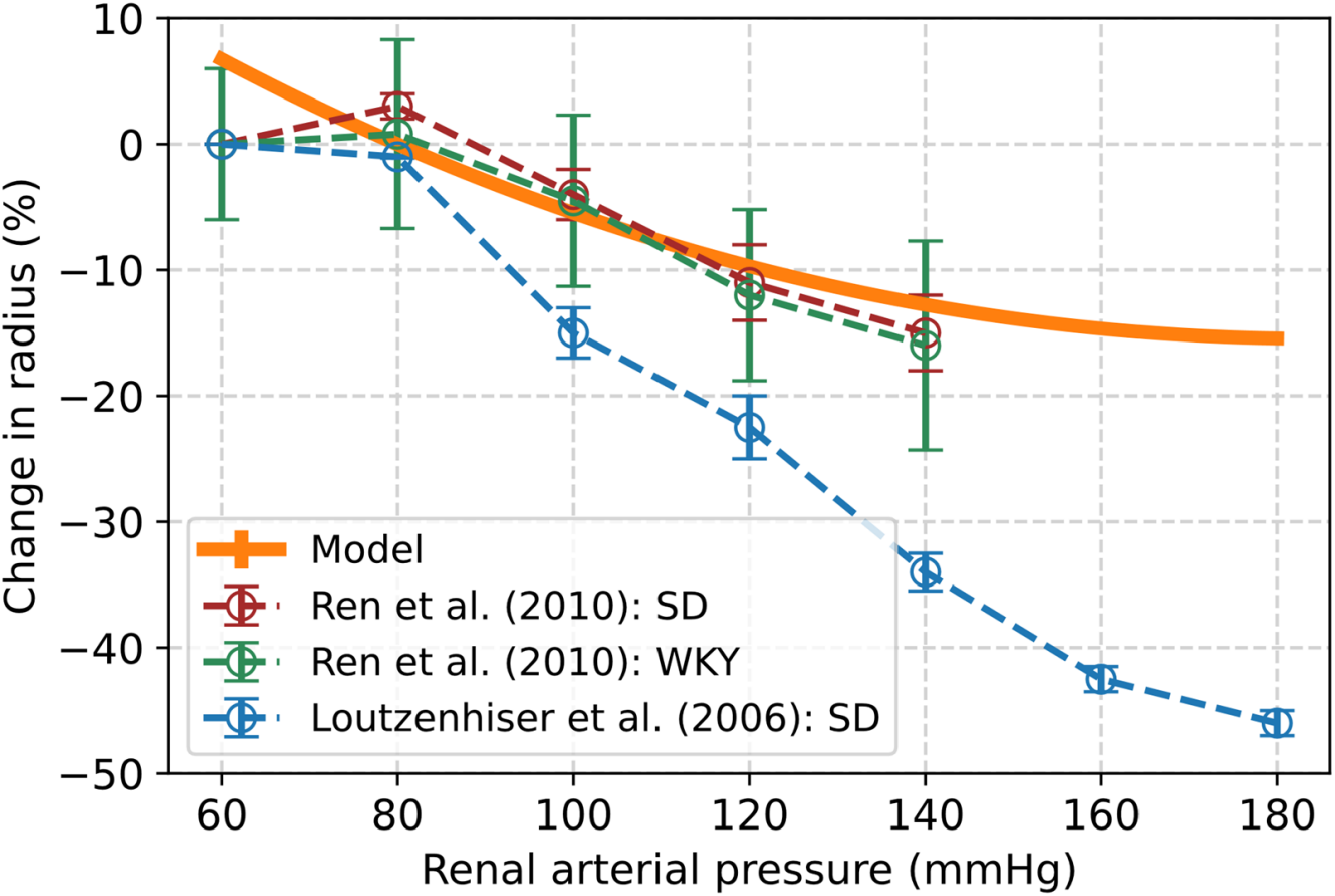
Change in renal arterial radius over a range of blood pressure as a result of the myogenic response in different strains of rats observed by (Loutzenhiser et al., 2002; Ren et al., 2010) and our myogenic response model. WKY, Wistar–Kyoto; SD, Sprague–Dawley.

The response of our modeled afferent diameter was compared with two different studies for validation. The study of Ren et al. observed the change in renal afferent diameter in isolated renal afferent arterioles with an intact glomerulus, obtained from male WKY‐rats (*n* = 5) and male Sprague–Dawley (SD) rats (*n* = 6). Loutzenhiser et al. used in vitro perfused hydronephrotic SD rat kidneys (*n* = 49). A comparison of our model over a renal arterial pressure range of 80 to 180 mmHg showed that our simulated results were in agreement with the incremental data reported by Ren et al. (mean deviation of 3%), Figure 4. The observations of Loutzenhiser et al. were less in line with our model results (mean deviation of 16%) (Loutzenhiser et al., 2002; Ren et al., 2010).

The modeled responses of RBF and the renal afferent arteriole were compared to three different studies. Walker et al. induced a step‐increase in renal arterial pressure from 100 to 148 mmHg and measured the change in afferent arteriolar diameter and blood flow in vitro in blood‐perfused isolated juxtamedullary nephrons obtained from SD rats (*n* = 11). Measurements were performed with an intact TGF system and after interruption of distal tubular flow by papillectomy (TGF independent). Takenaka et al. induced a step‐increase in renal arterial pressure, first from 101 to 123 mmHg and then from 123 to 148 mmHg. The change in afferent arteriolar diameter and blood flow is measured in vitro in blood‐perfused juxtamedullary nephrons obtained from SD rats (*n* = 9). Measurements were performed with intact TGF system and after furosemide injection and papillectomy (TGF‐independent). Just et al. increased blood pressure from 94 to 110 mmHg and measured renal vascular resistance and renal blood flow in vivo in male SD rats (*n* = 19). Measurements were performed with an intact TGF system and after furosemide injection (TGF‐independent). The results for the different TGF‐independent experimental set‐ups were similar (Just & Arendshorst, 2003; Walker et al., 2000; Takenaka et al., 1994).

**FIGURE 5:**
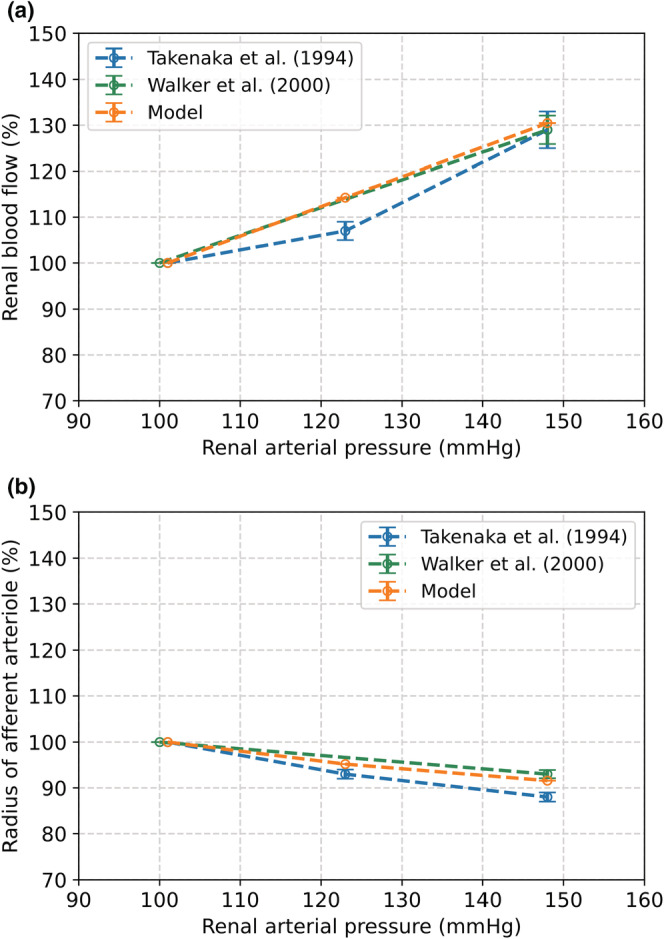
The effect of an increase in renal arterial blood pressure from 100 to 148 mmHg as a result from the MR model compared to observations by Walker et al. and Takenaka et al. (Walker et al., 2000; Takenaka et al., 1994). (a) Change in renal blood flow. (b) Change in radius of the afferent arteriole.

**FIGURE 6:**
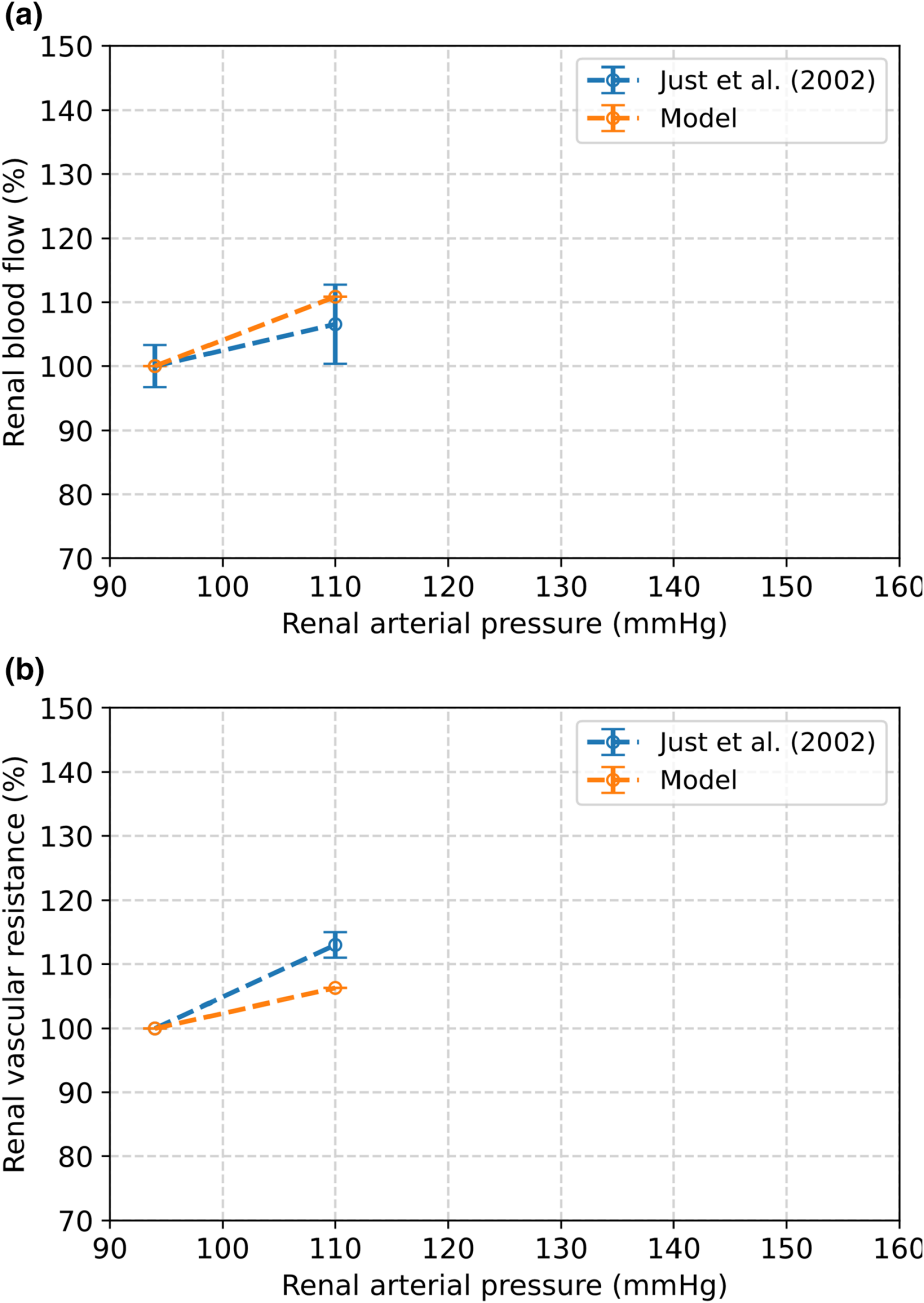
The effect of an increase in renal arterial blood pressure from 100 to 110 mmHg as a result from the MR model compared to observations by Just et al. (Just & Arendshorst, 2003). (a) Change in renal blood flow. (b) Change in renal vascular resistance.

Figure 5 shows that the changes in RBF and renal afferent arteriolar radius of our model were in line with the observations by Walker et al., as both values deviated by 2% after an increase in renal arterial pressure from 100 to 148 mmHg (Walker et al., 2000). Figure 5 also shows our MR model results compared to the experimental results of Takenaka et al. (Takenaka et al., 1994) After increasing blood pressure from 100 to 123 mmHg, the modeled change in RBF and afferent radius deviated by 7% and 2% from the observations by Takenaka et al., respectively. When blood pressure is increased to 148 mmHg, these deviations are 1% (RBF) and 4% (radius). Our model was also in line with observations by Just et al. at renal arterial pressures of 94 and 110 mmHg (Figure 6) (Just & Arendshorst, 2003). The modeled RBF and increase in renal vascular resistance deviated by 4% and 7% from the observations by Just et al., respectively.

#### 3.2.2 Tubuloglomerular feedback

**FIGURE B3:**
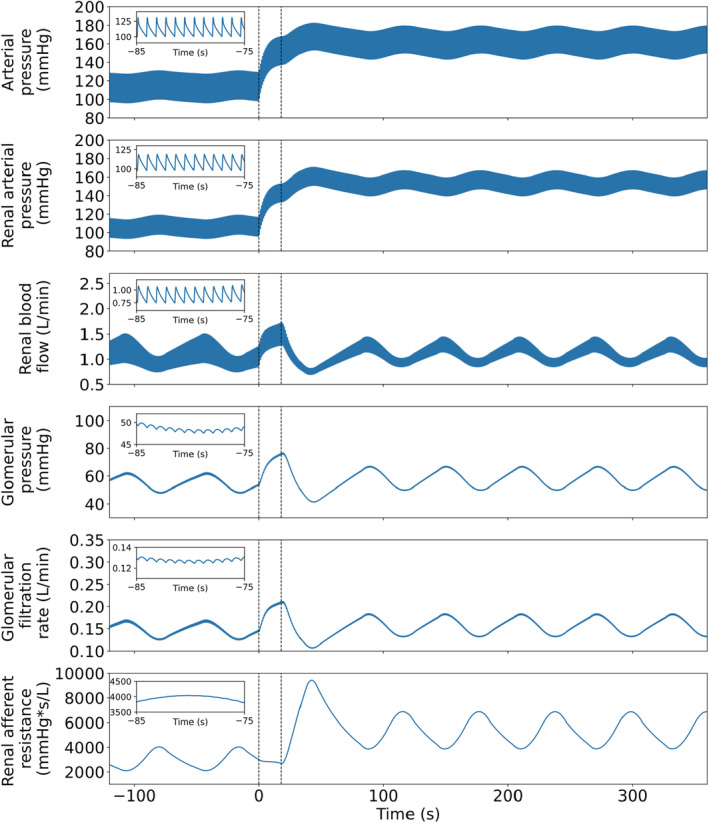
The dynamic behavior of renal arterial pressure, glomerular pressure, renal blood flow, glomerular filtration, and renal afferent resistance with renal autoregulation by the MR and TGF. At time *t* = 0 s (first dashed line), mean renal arterial pressure is elevated from 100 to 148 mmHg. Renal afferent resistance is increased by the myogenic response and tubuloglomerular feedback. TGF becomes active at *t* = 18 s (second dashed line). The subplots for the flows and pressures show small oscillations in these variables which are caused by heart rate.

Based on previous data on rats, the time constants were set to $\tau_{3}$ = 15 s and $\tau_{4}$ = 33 s and the delay *δ*_*3*_ = 18 s (Daniels & Arendshorst, 1990; Holstein‐Rathlou & Marsh, 1990), where the operating point was set to *op*_*GFR*_ = 149 ml/min (Spaanderman et al., 2000). *th*_*GFR*_ and *sa*_*GFR*_ were set to 144 ml/min and 333 ml/min, respectively (Boron & Boulpaep, 2012a; Rennke & Denker, 2006; Silverthorn, 2013). Response of renal pressures and flows to an increase in blood pressure and regulated by both the myogenic response and tubuloglomerular feedback can be found in Appendix B (Figure B3).

**FIGURE 7:**
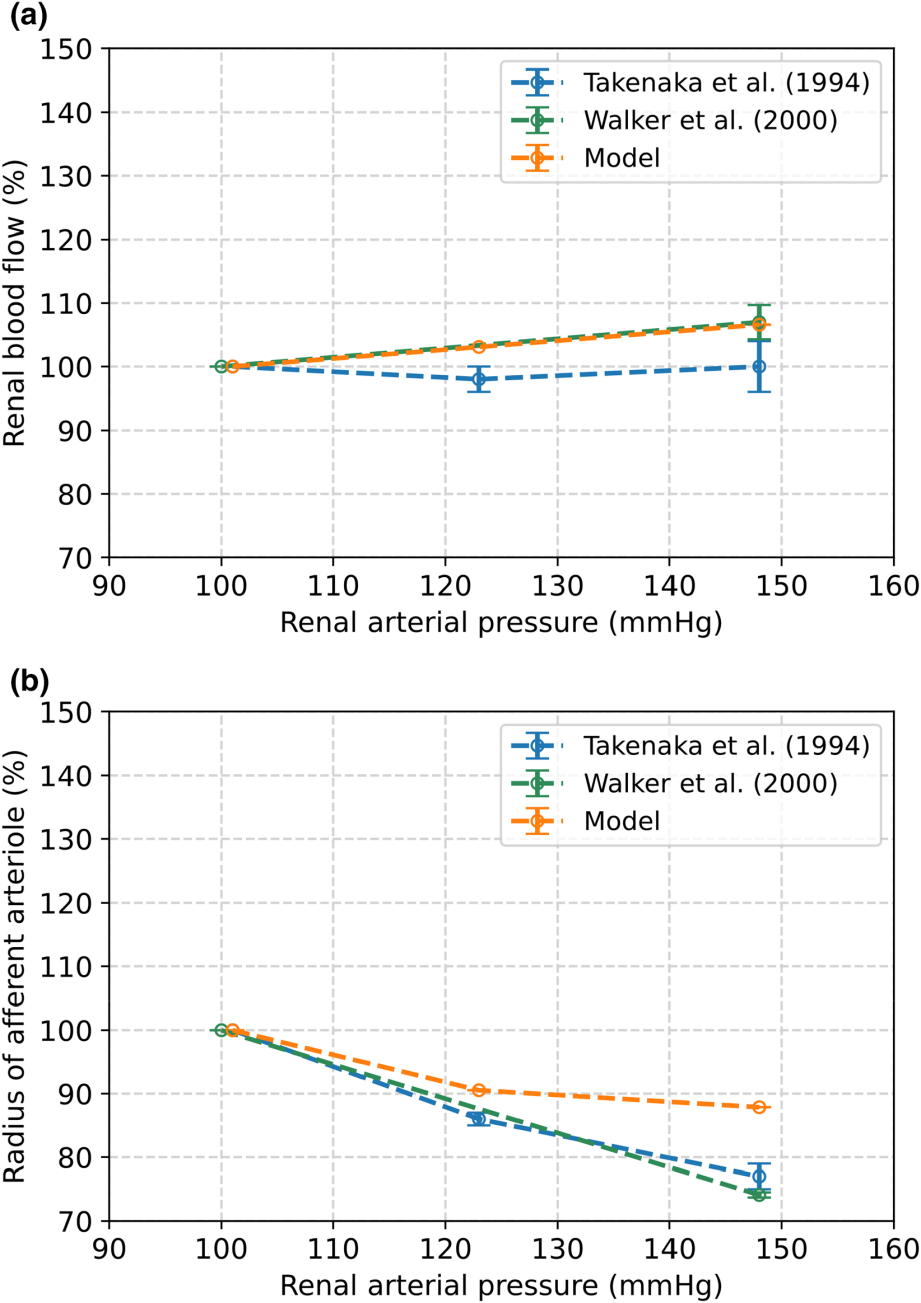
The effect of an increase in renal arterial blood pressure from 100 to 148 mmHg as a result from the complete autoregulation model compared to observations by Walker et al. and Takenaka et al. (Walker et al., 2000; Takenaka et al., 1994). (a) Change in renal blood flow. (b) Change in radius of the afferent arteriole.

Figure 7 presents the change in renal afferent radius and RBF of our model (MR and TGF) in response to an increase in renal blood pressure from 100 to mmHg in comparison to Walker et al. (2000). With a gain of *g*_*TGF*_ = 200, the RBF in our model changed according to the observations by Walker et al. Therefore, subsequent validation was performed with this gain.

**FIGURE 8:**
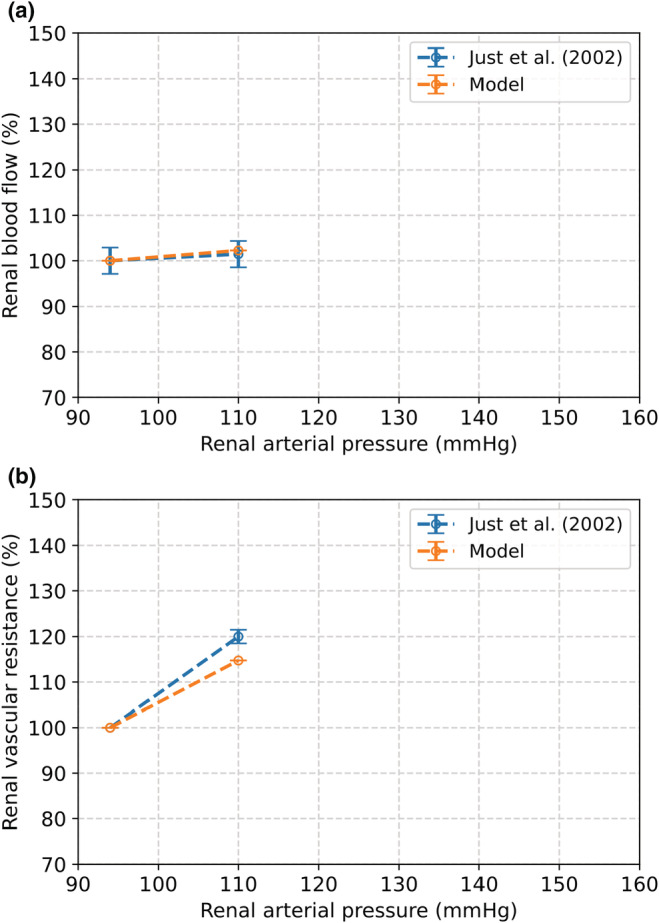
The effect of an increase in renal arterial blood pressure from 100 to 110 mmHg as a result from the complete autoregulation model compared to observations by Just et al. (Just & Arendshorst, 2003). (a) Change in renal blood flow. (b) Change in renal vascular resistance.

Figure 7 also shows the model results compared to the results of Takenaka et al. (Takenaka et al., 1994). After an increase in renal pressure from 100 to 123 mmHg, the modeled change in RBF and afferent radius both deviated by 5% from the results published by Takenaka et al., respectively. When blood pressure is increased to 148 mmHg, these deviations are 7% (RBF) and 11% (radius). The results of our model compared to Just et al. are depicted in Figure 8 (A: change in RBF, B: change in renal vascular resistance) (Just & Arendshorst, 2003). Our model showed a very good outcome in response to an increase in renal arterial pressure from 94 to 110 mmHg, as the increase in RBF deviated by less than 1% and the increase in renal vascular resistance by 5% compared to these changes observed in the rat experiments.

#### 3.2.3 Sensitivity analysis

Over a blood pressure increase of 100 to 123 and 148 mmHg, decreasing or increasing parameter *k* by 10% or 20% did not affect the results of the MR model by more than 5% and 9%, respectively. Over a blood pressure increase of 94 to 110 mmHg, decreasing or increasing parameter *k* by 10% or 20% did not affect the results of the MR model by more than 4% and 6%, respectively.

The results of the complete autoregulation model evaluated over a blood pressure increase of 100 to 123 and 148 mmHg were not affected by changing parameter *k* by 10% and 20%. Over a blood pressure increase from 94 to 110 mmHg, changing parameter *k* by 10% and 20% did also not affect the model results.

**FIGURE C1:**
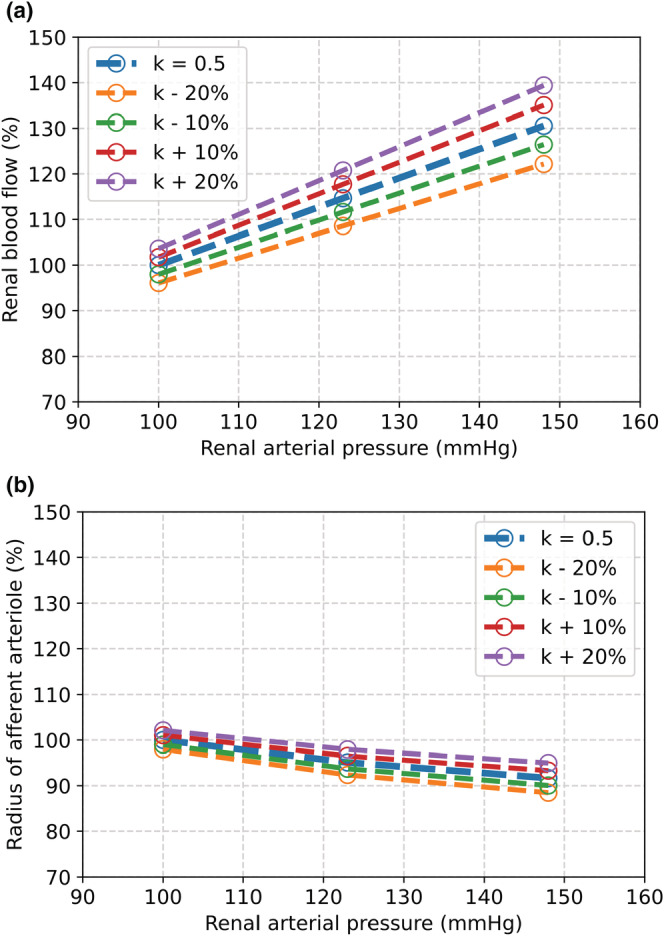
The effect of decreasing and increasing parameter *k* by 10% and 20% on the results of the MR model evaluated over an increase in blood pressure from 100 to 123 and 148 mmHg. (a) Change in renal blood flow. (b) Change in radius of the afferent arteriole.

**FIGURE C2:**
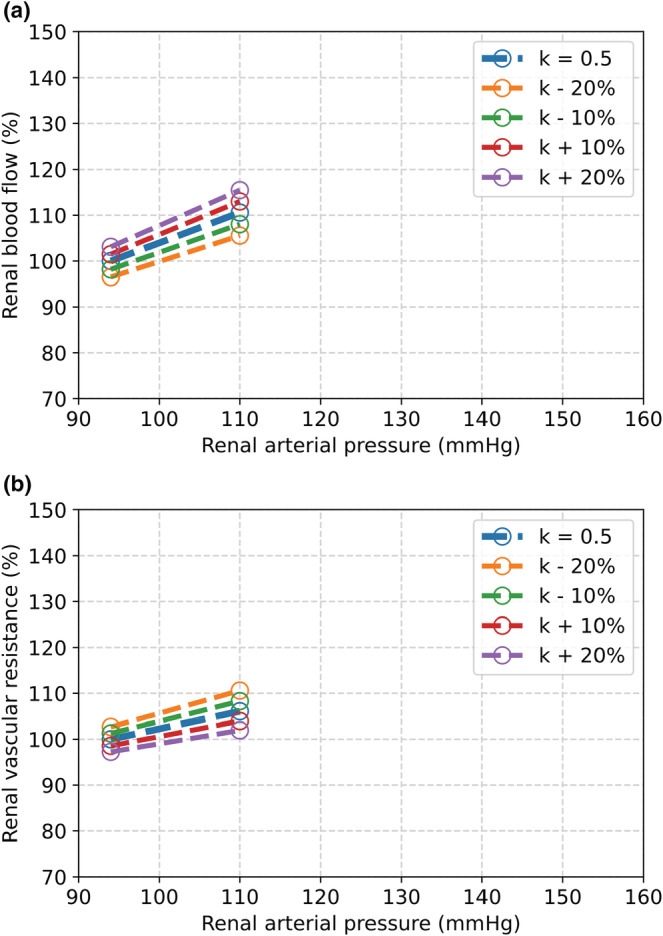
The effect of decreasing and increasing parameter *k* by 10% and 20% on the results of the MR model evaluated over an increase in blood pressure from 94 to 110 mmHg. (a) Change in renal blood flow. (b) Change in renal vascular resistance.

**FIGURE C3:**
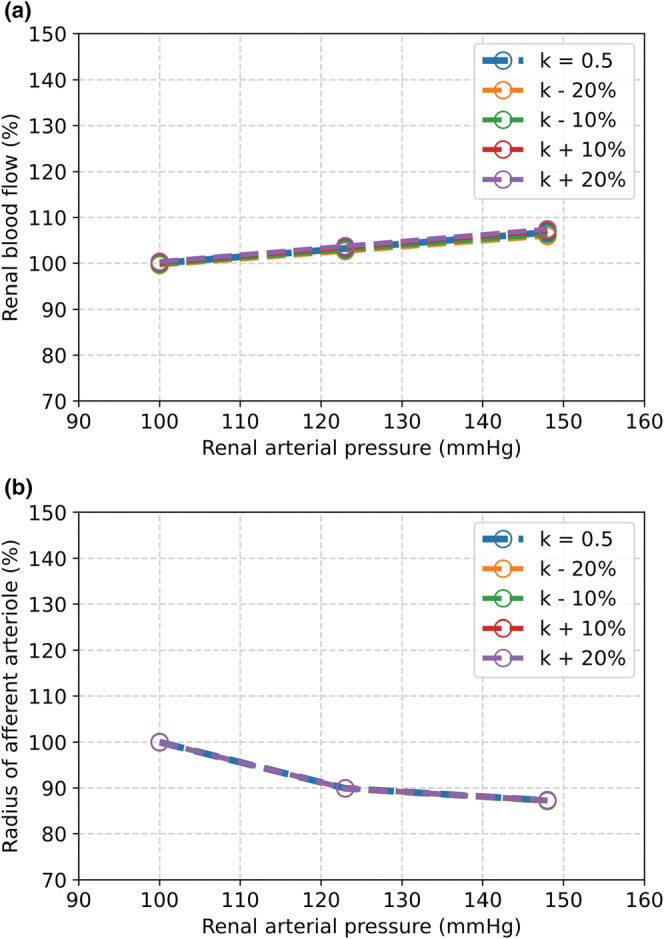
The effect of decreasing and increasing parameter *k* by 10% and 20% on the results of the complete renal autoregulation evaluated over an increase in blood pressure from 100 to 123 and 148 mmHg. (a) Change in renal blood flow. (b) Change in radius of the afferent arteriole.

**FIGURE C4:**
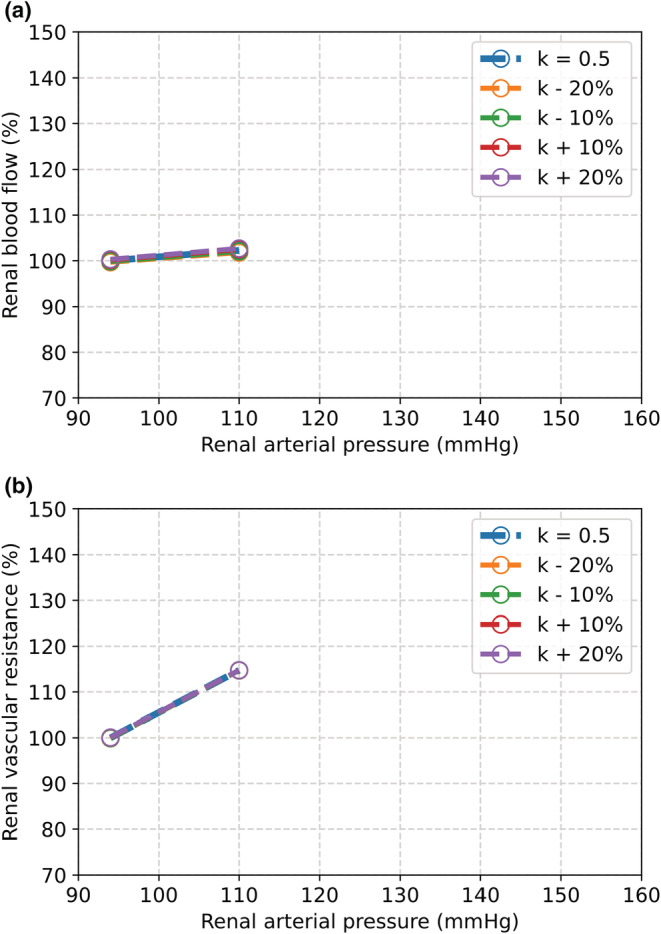
The effect of decreasing and increasing parameter *k* by 10% and 20% on the results of the complete renal autoregulation evaluated over an increase in blood pressure from 94 to 110 mmHg. (a) Change in renal blood flow. (b) Change in renal vascular resistance.

**FIGURE C5:**
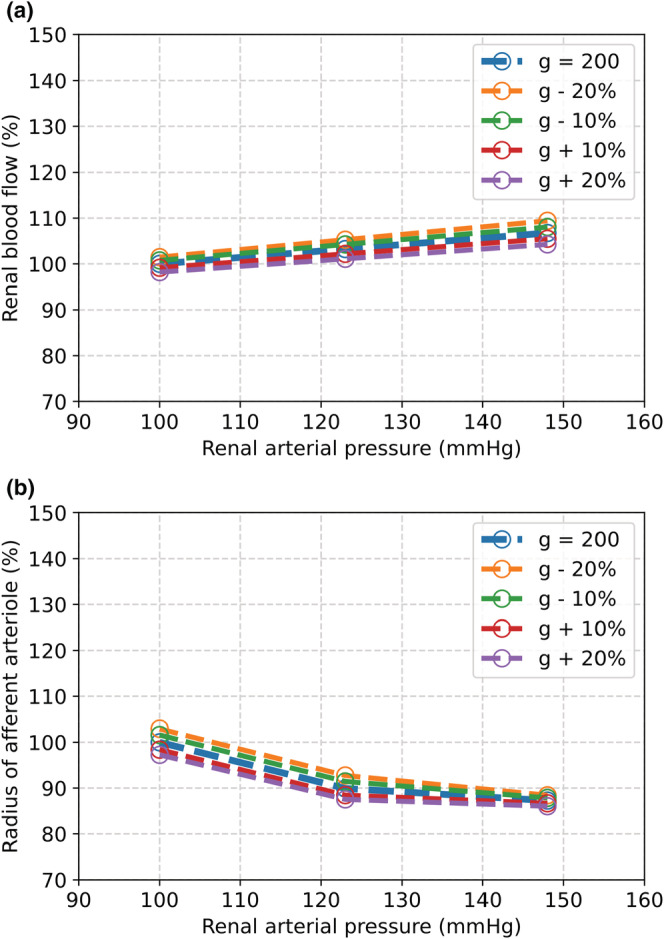
The effect of decreasing and increasing parameter *g*_*TGF*_ by 10% and 20% on the results of the complete renal autoregulation evaluated over an increase in blood pressure from 100 to 123 and 148 mmHg. (a) Change in renal blood flow. (b) Change in radius of the afferent arteriole.

**FIGURE C6:**
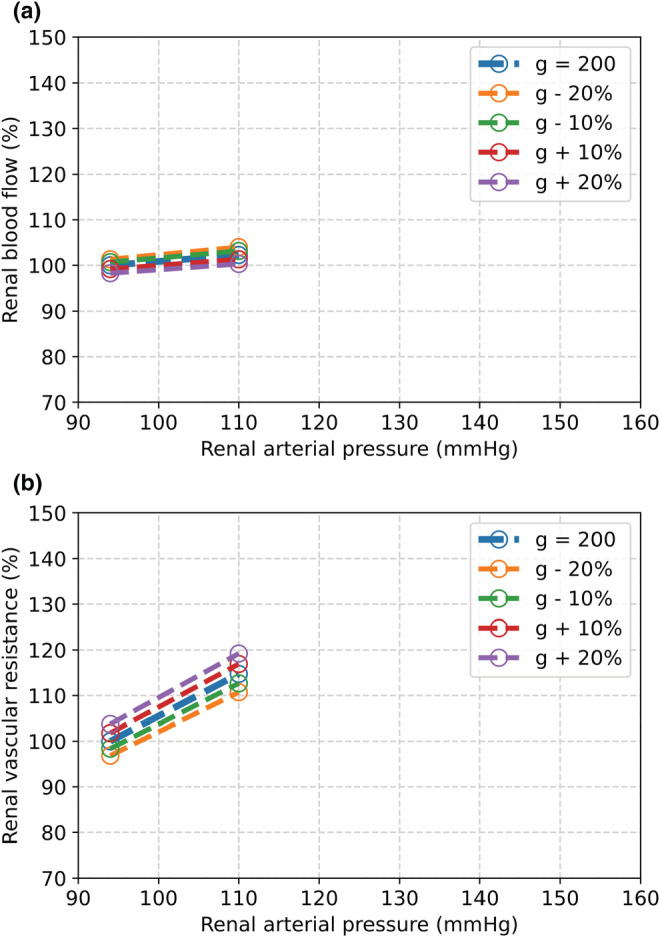
The effect of decreasing and increasing parameter *g*_*TGF*_ by 10% and 20% on the results of the complete renal autoregulation evaluated over an increase in blood pressure from 94 to 110 mmHg. (a) Change in renal blood flow. (b) Change in renal vascular resistance.

Over a blood pressure increase of 100 to 123 and 148 mmHg, decreasing or increasing *g*_*TGF*_ by 10% or 20% did not affect the results of the complete autoregulation model by more than 3% and 6%, respectively. Over a blood pressure increase of 94 to 110 mmHg, decreasing or increasing *g*_*TGF*_ by 10% or 20% did not affect the results of the renal autoregulation model by more than 2% and 3%, respectively. Additional figures can be found in Appendix C (Figure C1, C2, C3, C4, C5, C6).

## 4 DISCUSSION

During pregnancy, the maternal cardiovascular system goes through tremendous changes, which include changes in renal autoregulatory and functional physiology. We developed a renal autoregulation model implemented in a first‐trimester hemodynamic model that performs in line with values measured by others. Also, the model‐based changes in renal blood flow and resistance induced by an increase in blood pressure correspond to published observations.

The level of activation of the renal myogenic response depends on changes in the systemic blood pressure. We estimated the parameters for our MR model based on reported measurements (Hayashi et al., 1989; Spaanderman et al., 2000). We compared our modeled radius of the afferent arteriole to the radius in rats observed by different researchers (Figure 4) (Loutzenhiser et al., 2002; Ren et al., 2010). Our results were in agreement with the measurements by Ren et al., but Loutzenhiser et al. observed a different response of the afferent arteriole. The discrepancy between these measurements may be caused by the differences in experimental set‐up and used rat strains (van Drongelen et al., 2014). The response of the myogenic model was further validated based on the change in RBF, afferent radius, and resistance induced by a step increase in blood pressure. The largest deviation of our model compared to the literature was 7%. According to our limits set in advance, this implies that the results of our MR model are very good.

The TGF mechanism tends to maintain GFR constant over a broad range of blood pressures. It has been shown that there is a relation between GFR and the level of activation of TGF. In our study, we modeled the TGF by means of an activation function, for which we had to estimate the gain. To that end, the increases in RBF and radius induced by a blood pressure step were evaluated with different gains. With a gain of *g*_*TGF*_ = 200, our modeled RBF behaved in response to an increase in renal arterial pressure similar to the observations made by Walker et al. (2000). With the gain fixed at this value, we evaluated the complete renal autoregulation model by comparing our results to observations by Just et al. and Takenaka et al. (Just & Arendshorst, 2003; Takenaka et al., 1994). In response to an increase in renal arterial pressure, our model showed comparable changes in RBF, renal resistance and afferent radius. The above suggests that our renal autoregulation model is able to simulate the effect of a blood pressure alteration on RBF and the change in resistance.

Other models of renal autoregulation have been proposed (Aukland & Oien, 1987; Cupples et al., 1990; Holstein‐Rathlou & Marsh, 1990; Lush & Fray, 1984; Marsh et al., 2005; Pitman & Layton, 1989; Sgouralis & Layton, 2015). These mathematical models tend to focus more comprehensively on the tubular role in renal autoregulation, describing the influence of intratubular NaCl concentration at the site of the macula densa on the afferent arteriole. Since a change in intratubular NaCl concentration is related to a change in GFR and our hemodynamic model does not yet contain NaCl concentrations, we chose the GFR as input for our TGF model (Boron & Boulpaep, 2012a; Briggs & Schnermann, 1987). For this purpose, we used a piecewise linear activation curve. The use of an activation curve with an operating point is also described by Layton et al., however, they used a sigmoidal relationship between intratubular flow and intratubular Cl^−^ concentration to describe the TGF system (Sgouralis & Layton, 2015). Furthermore, other studies proposed a more extensive description of contraction of the afferent arteriole, including a mathematical description of calcium influx involved in the contraction of vascular smooth muscle cells (Lush & Fray, 1984; Marsh et al., 2005; Pitman & Layton, 1989). Another notable difference with earlier proposed models is that these solely represent the renal vasculature and non‐pregnant hemodynamics, whereas our renal autoregulation model is implemented in a greater whole‐body hemodynamic gestational model. Our renal autoregulation model was developed with the main focus on the hemodynamic properties of the MR and TGF. Therefore, we decided to keep our model transparent and robust, and model the kidneys as a lumped nephron, consisting of a glomerulus and tubule.

### 4.1 Strengths and limitations

In this study, we designed and implemented the renal myogenic response and tubuloglomerular feedback, the most relevant subsystems for renal autoregulation, in a mathematical first‐trimester hemodynamic model. The model used in this study is a lumped model consisting of 19 compartments, which means that the overall circulation is simplified but taken into account. The outcome of this model corresponds to normal physiological behavior as observed by others.

Despite this strength, we would like to address a few limitations of interest. First, it is important to realize that the model parameters, meaning the elastances, (unstressed) volumes, and resistances, were estimated to obtain physiological hemodynamics comparable to first‐trimester pregnancy. Even though the hemodynamic results of our model are comparable to values described in previous studies, additional empirical data may fortify the robustness of used values. Nonetheless, for our renal autoregulation model, the sensitivity analysis indicates that a small uncertainty in the model parameters does not affect our model results to a very large extent.

Second, only limited data were available for model validation. The model parameters for the hemodynamic model were derived from data obtained in pregnant women, with the goal to reach blood flows and pressures in a stable physiologic state. Data used for estimation of the model parameters and validation concerning the renal autoregulation model were obtained from male rats. Their response might differ from pregnant, female rats and more specifically pregnant humans. However, TGF is not suppressed during pregnancy but reset to operate under a sustained elevated GFR compared to non‐pregnant individuals (Reckelhoff et al., 1992). Other studies support this hypothesis, which states that despite renal vasodilation during pregnancy, renal autoregulation is still preserved (Baylis, 1994; Baylis & Blantz, 1985; Woods et al., 1987). Therefore, we think that our model is suitable to simulate renal autoregulation and may be used for research in pregnancy.

Third, we did not include the effect of the TGF on the efferent arteriole. The reason for this is that there is still quite some debate about the role of the efferent arteriole in renal autoregulation. Some studies suggest a role of the efferent arteriole in regulation of the GFR, although it is generally accepted that the afferent arteriole has a significantly greater impact in renal autoregulation (Blantz & Tucker, 1981; Davis, 1991; Ichikawa, 1982; Kleinstreuer, 2009; Ren et al., 2001). The renin–angiotensin–aldosterone system (RAAS) however does affect renal efferent arteriolar resistance (Carey & Siragy, 2003; Guyton & Hall, 2006; Ito & Abe, 1997), but it was beyond the scope of our study to include this influence in our model.

Fourth, glomerular filtration in our model is primarily determined by the pre‐ and post‐glomerular resistances which regulate renal blood flow and glomerular pressure. In reality, GFR is determined by the hydrostatic and colloid osmotic forces across the glomerular membrane and the filtration coefficient (Boron & Boulpaep, 2012a; Rennke & Denker, 2006; Silverthorn, 2013). We have not yet implemented the composition of blood in this model, and therefore are not yet able to account for the effect of the colloid osmotic pressure on GFR. As we only evaluate the effect of changes in blood pressure on renal resistance and hemodynamics, it can be assumed that colloid osmotic pressure remains constant and is accounted for by the glomerular resistance (*R*_*GL_TU*_). If colloid osmotic pressure is included in our model at a later stage, this resistance probably has to be altered to preserve a physiological GFR. Furthermore, reabsorption should then be modeled more realistically, as this process is currently also not yet included.

Fifth, it has to be kept in mind that besides renal autoregulation, other hemodynamic regulation mechanisms are not yet included. For instance, the previously mentioned RAAS affects efferent resistance and modulates both vascular tone and volume retention. Also, the baroreceptor reflex that lowers heart rate and vascular resistance in response to increased blood pressure and vice versa plays an important role in both renal and systemic sub‐acutely regulated hemodynamics (Boron & Boulpaep, 2012a; Boron & Boulpaep, 2012b). These two regulatory systems are not yet part of our gestational model, as in the current study, we chose to focus primarily on the fast renal autoregulation by MR and TGF. These autoregulatory mechanisms need to be in place before the relatively slow RAAS can be modeled, as local renal hemodynamics do influence the renin production. If other regulatory mechanisms were added to the model, the results would probably represent reality even better, but this was beyond the scope of the current research, as we primarily focused on rapid changes almost ultimately regulated by both studied mechanisms.

### 4.2 Clinical relevance and future perspectives

We developed a first‐trimester hemodynamic model that generates results in line with cardiovascular and renal physiology as described in the literature. Further development of our model is needed to simulate hemodynamics in early pregnancy even more realistically, such as implementing the RAAS, baroreceptor reflex, the sympathetic nervous system, urine production, and physiological composition of blood and urine (electrolytes and proteins). Our simulator model has the potential to be used for education or research purposes, but also to gain more clinical insight in the hemodynamic changes and complications during pregnancy and to simulate the effect on treatment regimens or preexisting disease states.

## 5 CONCLUSION

In conclusion, a mathematical description of the renal autoregulation was developed and implemented in a first‐trimester hemodynamic model. In a state of elevated blood pressure, the renal autoregulation model, consisting of the MR and TGF, shows similar behavior as has been described in literature. Further development should focus on extending the model with other regulatory mechanisms to understand the hemodynamic changes in healthy and complicated pregnancies.

## AUTHOR CONTRIBUTIONS

M.O., T.A.J., J.D, and B.E.W. conceived and designed research; M.O. performed experiments; M.O., T.A.J., J.D, B.E.W., and M.E.A.S. analyzed data; M.O., T.A.J., J.D, B.E.W., and M.E.A.S. interpreted results of experiments; M.O. prepared figures; M.O. drafted manuscript; M.O., J.D, B.E.W., and M.E.A.S. edited and revised manuscript; M.O., T.A.J., J.D, B.E.W. and M.E.A.S. approved final version of manuscript.

## Endnote

^1^
Equation 6 differs from the one described in the article by Williamson et al. (Williamson et al., 2008), as there was a discrepancy between the mathematical description and the corresponding autoregulatory curve. We thank Dr. Williamson for providing the correct equation on our request.

## APPENDIX A

**TABLE A1:** The input values of the unstressed volume (V_0_), starting volume (V), and elastance (E) for each compartment. Parameters are based on a woman with a body surface of 1.78 m^2^ (BioGears, 2018; Goodwin et al., 2004; van Meurs & Antonius, 2018)

Compartment	V_0_ (L)	V (L)	E (mmHg/L)
PA	0.050	0.186	133
PV	0.350	0.460	64.5
LA*	−0.005	0.047	138–360
LV*	0.014	0.121	77–3860
AA	0.068	0.135	1288
AD	0.175	0.333	539
UB	0.410	0.681	58.7
LB	1.614	2.295	23.3
AR	0.011	0.015	16,400
GL	0.020	0.027	1375
TU	0.018	0.024	1000
VR	0.013	0.024	1600
UA	0.210	0.280	1143
SA	0.060	0.080	3500
UV	0.400	0.500	64
PL	0.090	0.120	333
VC	0.956	1.197	12.9
RA*	0.009	0.050	73–400
RV*	0.045	0.182	26–333

* Time‐varying elastance.

**TABLE A2:** The input values for the resistances of each connector (BioGears, 2018; Goodwin et al., 2004; van Meurs & Antonius, 2018)

Connector	Resistance (mmHg*s/L)
PA_PV	105
PV_LA	3
AA_AD	20
AA_UB	2100
AD_LB	2000
AD_AR	200
AR_GL^∆^	1275
GL_TU°	15,470
GL_VR	3300
TU_VR°	4200
VR_VC	180
AD_UA	133
UA_SA	1013
SA_UV	74,400
UA_UV	34,615
SA_PL	5000
PL_UV	178
UV_VC	300
UB_VC	500
LB_VC	265
VC_RA	3
LA_LV^	3
LV_AA^	8
RA_RV^	3
RV_PA^	3

*Note*: ^∆^Baseline resistance; °No backflow; ^Valve.

**TABLE A3:** The target and resulting blood pressures in each compartment

Compartment	Pressure (mmHg) systolic/diastolic (mean arterial pressure)		
	Model value	Target value	Source
PA	23/13 (16)	8–25	(Boron & Boulpaep, 2012c; Guyton & Hall, 2016)
PV	9/7 (8)	10	(Boron & Boulpaep, 2012c; Guyton & Hall, 2016)
LA	10/6 (7)	8–10	(Boron & Boulpaep, 2012c; Guyton & Hall, 2016)
LV	108/5 (39)	10–120	(Boron & Boulpaep, 2012c; Guyton & Hall, 2016)
AA	102/71 (81)	65–120	(Cheung & Lafayette, 2013; Odutayo & Hladunewich, 2012; Spaanderman et al., 2000)
AD	94/71 (79)	65–120	(Avni et al., 2010; Cheung & Lafayette, 2013; Spaanderman et al., 2000)
UB	19/19 (19)	~17	(Guyton & Hall, 2016)
LB	13/13 (13)	~17	(Guyton & Hall, 2016)
AR	89/69 (76)	70–100	(Guyton & Hall, 2016; Thurau, 1964)
GL	57/54 (55)	50–60	(Boron & Boulpaep, 2012a; Silverthorn, 2013)
TU	17/17 (17)	18–20	(Guyton & Hall, 2016; Thurau, 1964)
VR	7/7 (7)	8	(Guyton & Hall, 2016)
UA	86/72 (77)	80–100	(Clark et al., 2018; Wang & Zhao, 2010)
SA	69/64 (66)	~70	(Clark et al., 2018; Wang & Zhao, 2010)
UV	8/8 (8)	8	(Clark et al., 2018)
PL	10/10 (10)	10	(Clark et al., 2018)
VC	4/4 (4)	4/4	(Guyton & Hall, 2016)
RA	6/2 (3)	0–7	(Mebazaa et al., 2004)
RV	24/2 (9)	0–25	(Mebazaa et al., 2004)

**TABLE A4:** The target and resulting blood flows over each connector given by the model

Connector	Flow (L/min)		
	Model value	Target value	Source
PA_PV	5.776	5.7–6	(Meah et al., 2016; Spaanderman et al., 2000)
PV_LA	5.776	5.7–6	(Meah et al., 2016; Spaanderman et al., 2000)
AA_AD	3.956	4–4.2	CO – flow to UB
AA_UB	1.883	1.7–1.8	30%*CO (Zamboni et al., 2018)
AD_LB	2.057	2–2.2	70%*CO – RBF – uterine flow (Zamboni et al., 2018)
AD_AR	1.040	1.1	(Spaanderman et al., 2000)
AR_GL	1.040	1.1	(Spaanderman et al., 2000)
GL_TU	0.149	0.15	(Spaanderman et al., 2000)
GL_VR	0.890	0.95	(Spaanderman et al., 2000)
TU_VR	0.149	0.15	(Spaanderman et al., 2000)
VR_VC	1.040	1.1	(Spaanderman et al., 2000)
AD_UA	0.857	0.90	(Sipos et al., 2013; Wang & Zhao, 2010)
UA_SA	0.733	0.77	(Sipos et al., 2013; Wang & Zhao, 2010)
SA_UV	0.048	0.05	(Sipos et al., 2013; Wang & Zhao, 2010)
UA_UV	0.124	0.13	(Sipos et al., 2013; Wang & Zhao, 2010)
SA_PL	0.685	0.6–0.7	(Sipos et al., 2013; Wang & Zhao, 2010)
PL_UV	0.685	0.6–0.7	(Sipos et al., 2013; Wang & Zhao, 2010)
UV_VC	0.857	0.9	(Sipos et al., 2013; Wang & Zhao, 2010)
UB_VC	1.883	1.7–1.8	30%*CO (Zamboni et al., 2018)
LB_VC	2.057	2–2.2	70%*CO – RBF – uterine flow (Zamboni et al., 2018)
VC_RA	5.776	5.7–6	(Meah et al., 2016; Spaanderman et al., 2000)
LA_LV	5.776	5.7–6	(Meah et al., 2016; Spaanderman et al., 2000)
LV_AA	5.776°	5.7–6	(Meah et al., 2016; Spaanderman et al., 2000)
RA_RV	5.776	5.7–6	(Meah et al., 2016; Spaanderman et al., 2000)
RV_PA	5.776	5.7–6	(Meah et al., 2016; Spaanderman et al., 2000)

*Note*: °Equals cardiac output (CO).
